# Source Tracking Based on Core Genome SNV and CRISPR Typing of *Salmonella enterica* Serovar Heidelberg Isolates Involved in Foodborne Outbreaks in Québec, 2012

**DOI:** 10.3389/fmicb.2020.01317

**Published:** 2020-06-17

**Authors:** Khadidja Yousfi, Valentine Usongo, Chrystal Berry, Rufaida H. Khan, Denise M. Tremblay, Sylvain Moineau, Michael R. Mulvey, Florence Doualla-Bell, Eric Fournier, Celine Nadon, Lawrence Goodridge, Sadjia Bekal

**Affiliations:** ^1^Laboratoire de Santé Publique du Québec, Institut National de Santé Publique du Québec, Sainte-Anne-de-Bellevue, QC, Canada; ^2^Department of Food Science and Agricultural Chemistry, McGill University, Sainte-Anne-de-Bellevue, QC, Canada; ^3^National Microbiology Laboratory, Public Health Agency of Canada, Winnipeg, MB, Canada; ^4^Département de Biochimie, de Microbiologie et de Bio-Informatique, Faculté des Sciences et de Génie, Université Laval, Quebec City, QC, Canada; ^5^Département de Microbiologie, Infectiologie et Immunologie, Université de Montréal, Montreal, QC, Canada

**Keywords:** source attribution, *Salmonella enterica* serovar Heidelberg, core genome SNV, CRISPR, PFGE, foodborne outbreaks, genomic typing

## Abstract

Whole-genome sequencing (WGS) is the method of choice for bacterial subtyping and it is rapidly replacing the more traditional methods such as pulsed-field gel electrophoresis (PFGE). Here we used the high-resolution core genome single nucleotide variant (cgSNV) typing method to characterize clinical and food from *Salmonella enterica* serovar Heidelberg isolates in the context of source attribution. Additionally, clustered regularly interspaced short palindromic repeats (CRISPR) analysis was included to further support this method. Our results revealed that cgSNV was highly discriminatory and separated the outbreak isolates into distinct clusters (0–4 SNVs). CRISPR analysis was also able to distinguish outbreak strains from epidemiologically unrelated isolates. Specifically, our data clearly demonstrated the strength of these two methods to determine the probable source(s) of a 2012 epidemiologically characterized outbreak of *S.* Heidelberg. Using molecular cut-off of 0–10 SNVs, the cgSNV analysis of 246 clinical and food isolates of *S.* Heidelberg collected in Québec, in the same year of the outbreak event, revealed that retail and abattoir chicken isolates likely represent an important source of human infection to *S*. Heidelberg. Interestingly, the isolates genetically related by cgSNV also harbored the same CRISPR as outbreak isolates and clusters. This indicates that CRISPR profiles can be useful as a complementary approach to determine source attribution in foodborne outbreaks. Use of the genomic analysis also allowed to identify a large number of cases that were missed by PFGE, indicating that most outbreaks are probably underestimated. Although epidemiological information must still support WGS-based results, cgSNV method is a highly discriminatory method for the resolution of outbreak events and the attribution of these events to their respective sources. CRISPR typing can serve as a complimentary tool to this analysis during source tracking.

## Introduction

Non-typhoidal *Salmonella* (NTS) *enterica* serovars are the most important causes of bacterial gastroenteritis (Acheson and Hohmann, 2001; Rabsch et al., 2001). Globally, it has been estimated that each year, approximately 93.8 million cases and 155,000 deaths are attributable to NTS (Majowicz et al., 2010). In Canada for example, NTS cause an estimated 88,000 gastrointestinal infections each year (Thomas et al., 2013). Among the NTS serovars, *Salmonella enterica* serovar Heidelberg is ranked amongst the top three serovars isolated from humans infected with *Salmonella* in Canada (National Enteric Surveillance Program [NESP], 2018). Outbreaks involving *S*. Heidelberg have been linked to the consumption of poultry and poultry products (Antunes et al., 2016). During epidemiological investigations, identifying the source(s) of foodborne outbreaks is important in order to implement corrective measures in the food chain that would prevent the reoccurrence of such outbreaks. Pulsed-field gel electrophoresis (PFGE) has been the Gold Standard method used by PulseNet Canada (PNC) since early the 2000s for the molecular typing of foodborne pathogens including *Salmonella* during outbreak investigations. However, a major drawback with the use of PFGE during outbreak investigations is its low resolution power that is further exacerbated when applied to *S*. Heidelberg typing owing to the extremely low genetic diversity of this serovar (Bekal et al., 2016; Vincent et al., 2018). This lack of adequate discriminatory power makes it difficult to track the source of a specific clone of *S*. Heidelberg implicated in foodborne outbreaks. Whole genome sequence (WGS) based methods, owing to their growing availability and high genomic resolution, are rapidly replacing traditional typing methods such as PFGE within major public health laboratories including PulseNet Canada (PNC) (Nadon et al., 2017). WGS-based methods include the high resolution core genome single nucleotide variant analysis typing method (cgSNV). The utility of this typing method in surveillance and outbreak detection has been already demonstrated in several *Salmonella* serovars in Canada, United States, and Australia (Hoffmann et al., 2014; Fu et al., 2017; Nadon et al., 2017). WGS data can also be mined for the presence of clustered regularly interspaced short palindromic repeats (CRISPR) arrays. CRISPR is part of an adaptive bacterial immunity system that precisely targets invading genetic elements such as phage genomes and plasmids (Garneau et al., 2010). Specifically, a CRISPR array is a genetic structure found in many bacterial genomes that consists of short repeat sequences spaced by short non-repetitive variable sequences named spacers. Variation in spacer content has been exploited for bacterial subtyping and epidemiological investigations in major *Salmonella* serovars (Shariat and Dudley, 2014).

Here, we assessed the effectiveness of the combination of cgSNV and CRISPR typing for the source tracking of an epidemiologically well-characterized foodborne outbreaks of *S.* Heidelberg that occurred in Québec in 2012 and non-documented cases. We also wanted to determine whether CRISPR evolution had any impact on the fitness of these isolates and also whether this evolution correlated with that of the cgSNV.

## Materials and Methods

### Bacterial Isolates Sources

A total of 246 *S.* Heidelberg isolates from Québec were included in the study. Identification and serotyping were confirmed by the standardized conventional agglutination and PFGE protocols following the PulseNet Canada (PNC) guidelines. One hundred ninety-three clinical isolates were also obtained from patients in Quebec hospitals as part of the active provincial surveillance program. Two food isolates (14-2571 and 14-2570) were obtained during the food poisoning incidents reported by the Ministère de l’Agriculture, des Pêcheries et de l’Alimentation du Québec (MAPAQ). Two outbreaks epidemiologically well documented were included: outbreak 2012-04-SH (*n* = 6 human isolates) and outbreak 2012-05-SH (*n* = 8 human isolates and *n* = 2 food isolates). In this study, non-documented cases (NDC) refer to isolates with incomplete epidemiological data.

No food isolates were identified during the investigation of the outbreak 2012-04-SH. Amongst the 193 human isolates, 155 (80.3%) isolates exhibited pulsotype 2 (PNC designation SHXAI.0001/SHBNI.0001) which represented more than 50% of Quebec clinical isolates, in 2012. The other pulsotypes were used as external controls.

Fifty-one food and environmental isolates were collected as part of the Canadian Integrated Program for Antimicrobial Resistance Surveillance (CIPARS). Abattoir sampling was performed from cecal contents taken post-slaughter from broiler chickens. Routine food surveillance of *Salmonella* was performed on chicken and turkey and the samples were collected from chain stores and independent butchers.

These 51 *S*. Heidelberg isolates were subdivided as: chicken samples (n = 23), prepackaged chicken samples (*n* = 13), turkey samples (*n* = 7), and cecal chicken samples (*n* = 8). Epidemiological and genomic data of the isolates recovered from food and environmental samples were previously documented (Edirmanasinghe et al., 2017). Table 1 summarizes the metadata of the 246 *S*. Heidelberg isolates analyzed in this study.

**TABLE 1:** Epidemiologic and subtyping results of 246 *S*. Heidelberg human clinical and food isolates used in this study.

Cluster	ST	Isolate No.	Source	Isolation date by month and year	Outbreak code/food type	Pulsotype in Québec	Phage type	CRISPR Profile	PulseNet Canada XbaI and BlnI PFGE pattern designation
1	7	12-1195	Human	02-2012	NDC	2	29	CP1	SHEXAI.0001/SHEBNI.0001
1	9	12-1667	Human	02-2012	NDC	2	19	CP1	SHEXAI.0001/SHEBNI.0001
1	11	12-2458	Human	03-2012	NDC	2	29	CP1	SHEXAI.0001/SHEBNI.0001
1	11	ID116897	Human	03-2012	NDC	2	29	CP1	SHEXAI.0001/SHEBNI.0001
1	14	12-2694	Human	04-2012	NDC	2	Atypical	CP1	SHEXAI.0001/SHEBNI.0001
1	14	ID116816	Human	03-2012	NDC	2	53	CP1	SHEXAI.0001/SHEBNI.0001
1	14	N13-01307	NA	03-2012	Prepackaged chicken	2	NA	CP1	SHEXAI.0001/SHEBNI.0001
1	14	N13-01308	NA	03-2012	Prepackaged chicken	2	NA	CP1	SHEXAI.0001/SHEBNI.0001
1	16	12-3227	Human	05-2012	NDC	2	19	CP1	SHEXAI.0001/SHEBNI.0001
1	17	12-3330	Human	05-2012	NDC	2	19	CP1	SHEXAI.0001/SHEBNI.0001
1	17	ID117647	Human	05-2012	NDC	2	29	CP1	SHEXAI.0001/SHEBNI.0001
1	17	ID118194	Human	05-2012	NDC	2	19	CP1	SHEXAI.0001/SHEBNI.0001
1	18	12-3383	Human	2012	NDC	NA	NA	CP1	Not assigned
1	18	12-3458	Human	2012	NDC	NA	NA	CP1	Not assigned
1	18	12-3461	Human	2012	NDC	NA	NA	CP1	Not assigned
1	18	14-2562	Human	05-2012	05-2012-SH	2	19	CP1	SHEXAI.0001/SHEBNI.0001
1	18	14-2564	Human	05-2012	05-2012-SH	2	19	CP1	SHEXAI.0001/SHEBNI.0001
1	18	14-2565	Human	05-2012	05-2012-SH	2	19	CP1	SHEXAI.0001/SHEBNI.0001
1	18	14-2566	Human	05-2012	05-2012-SH	2	19	CP1	SHEXAI.0001/SHEBNI.0001
1	18	14-2567	Human	05-2012	05-2012-SH	2	19	CP1	SHEXAI.0001/SHEBNI.0001
1	18	14-2569	Human	05-2012	05-2012-SH	2	19	CP1	SHEXAI.0001/SHEBNI.0001
1	18	14-2571	Food	05-2012	05-2012-SH	2	NA	CP1	SHEXAI.0001/SHEBNI.0001
1	18	ID115637	Human	01-2012	NDC	2	19	CP1	SHEXAI.0001/SHEBNI.0001
1	18	ID117366	Human	04-2012	NDC	2	19	CP1	SHEXAI.0001/SHEBNI.0001
1	18	ID117813	Human	05-2012	NDC	2	19	CP1	SHEXAI.0001/SHEBNI.0001
1	18	ID117817	Human	05-2012	NDC	2	19	CP1	SHEXAI.0001/SHEBNI.0001
1	18	ID117828	Human	05-2012	NDC	2	19	CP1	SHEXAI.0001/SHEBNI.0001
1	18	ID118162	Human	05-2012	NDC	2	19	CP1	SHEXAI.0001/SHEBNI.0001
1	18	ID120183	Human	09-2012	NDC	2	19	CP1	SHEXAI.0001/SHEBNI.0001
1	18	N13-01322	NA	05-2012	Prepackaged chicken	2	NA	CP1	SHEXAI.0001/SHEBNI.0001
1	18	N13-01329	NA	05-2012	Chicken sample	2	NA	CP1	SHEXAI.0001/SHEBNI.0001
1	18	ID117324	Human	04-2012	NDC	2	19	CP1	SHEXAI.0001/SHEBNI.0001
1	18	ID115858	Human	01-2012	NDC	2	19	CP1	SHEXAI.0001/SHEBNI.0001
1	18	ID117099	Human	04-2012	NDC	2	19	CP1	SHEXAI.0001/SHEBNI.0001
1	18	ID116170	Human	02-2102	NDC	2	19	CP1	SHEXAI.0001/SHEBNI.0001
1	18	ID119006	Human	07-2012	NDC	2	19	CP1	SHEXAI.0001/SHEBNI.0001
1	18	ID117407	Human	04-2012	NDC	NA	NA	CP1	Not assigned
1	18	N13-01298	NA	01-2012	Chicken sample	2	NA	CP1	SHEXAI.0001/SHEBNI.0001
1	18	12-5444	Human	08-2012	NDC	2	29	CP1	SHEXAI.0001/SHEBNI.0001
1	18	12-3757	Human	05-2012	No NDC	2	29	CP1	SHEXAI.0001/SHEBNI.0001
1	18	12-7080	Human	09-2012	NDC	2	29	CP1	SHEXAI.0001/SHEBNI.0001
1	18	12-4374	Human	06-2012	NDC	4	41	CP1	SHEXAI.0011
1	18	12-4585	Human	07-2012	NDC	4	41	CP1	SHEXAI.0011
1	18	12-3755	Human	06-2012	NDC	4	41	CP1	SHEXAI.0011
1	21	12-4367Sa	Human	06-2012	NDC	2	19	CP1	SHEXAI.0001/SHEBNI.0001
1	21	ID116158	Human	02-2012	NDC	2	19	CP1	SHEXAI.0001/SHEBNI.0001
1	21	ID116758	Human	03-2012	NDC	2	19	CP1	SHEXAI.0001/SHEBNI.0001
1	21	ID117410	Human	04-2012	NDC	2	19	CP1	SHEXAI.0001/SHEBNI.0001
1	22	12-5152	Human	07-2012	NDC	2	29	CP1	SHEXAI.0001/SHEBNI.0001
1	34	14-2568	Human	05-2012	05-2012-SH	2	19	CP1	SHEXAI.0001/SHEBNI.0001
1	35	14-2570	Food	05-2012	05-2012-SH	2	NA	CP1	SHEXAI.0001/SHEBNI.0001
1	36	ID115636	Human	01-2012	NDC	2	19	CP1	SHEXAI.0001/SHEBNI.0001
1	36	ID115656	Human	01-2012	NDC	84	19	CP1	SHEXAI.0002
1	37	ID115663	Human	01-2012	NDC	2	19	CP1	SHEXAI.0001/SHEBNI.0001
1	39	ID115841	Human	01-2012	NDC	2	19	CP1	SHEXAI.0001/SHEBNI.0001
1	40	ID116003	Human	02-2012	NDC	2	19	CP1	SHEXAI.0001/SHEBNI.0001
1	42	ID116299	Human	02-2012	NDC	2	19	CP1	SHEXAI.0001/SHEBNI.0001
1	44	ID116464	Human	02-2012	NDC	2	17	CP1	SHEXAI.0001/SHEBNI.0001
1	45	ID116500	Human	03-2012	NDC	2	19	CP1	SHEXAI.0001/SHEBNI.0001
1	58	ID117689	Human	05-2012	NDC	2	19a	CP1	SHEXAI.0001/SHEBNI.0001
1	58	ID120014	Human	08-2012	NDC	2	19a	CP1	SHEXAI.0001/SHEBNI.0001
1	59	ID117794-m	Human	05-2012	05-2012-SH	2	19	CP1	SHEXAI.0001/SHEBNI.0001
1	61	ID117882	Human	05-2012	NDC	2	19	CP1	SHEXAI.0001/SHEBNI.0001
1	63	ID117991	Human	05-2012	NDC	2	19	CP1	SHEXAI.0001/SHEBNI.0001
1	76	ID118719	Human	06-2012	NDC	2	19	CP1	SHEXAI.0001/SHEBNI.0001
1	82	ID119109	Human	07-2012	NDC	2	19	CP1	SHEXAI.0001/SHEBNI.0001
1	83	ID119158	Human	07-2012	NDC	2	19	CP1	SHEXAI.0001/SHEBNI.0001
1	86	ID119477	Human	08-2012	NDC	2	19	CP1	SHEXAI.0001/SHEBNI.0001
1	87	ID119588	Human	08-2012	NDC	2	19	CP1	SHEXAI.0001/SHEBNI.0001
1	97	ID120223	Human	09-2012	NDC	2	17	CP1	SHEXAI.0001/SHEBNI.0001
1	97	ID120448	Human	09-2012	NDC	2	19	CP1	SHEXAI.0001/SHEBNI.0001
1	97	ID120450	Human	09-2012	NDC	2	36	CP1	SHEXAI.0001/SHEBNI.0001
1	98	ID120433	Human	09-2012	NDC	2	29	CP1	SHEXAI.0001/SHEBNI.0001
1	103	ID121112	Human	10-2012	NDC	2	19	CP1	SHEXAI.0001/SHEBNI.0001
1	103	ID121120	Human	10-2012	NDC	2	19	CP1	SHEXAI.0001/SHEBNI.0001
1	114	N13-01291	NA	01-2012	Chicken cecal	2	NA	CP1	SHEXAI.0001/SHEBNI.0001
1	116	N13-01293	NA	01-2012	Chicken sample	2	NA	CP1	SHEXAI.0001/SHEBNI.0001
1	118	N13-01296	NA	2012	Chicken sample	2	NA	CP1	SHEXAI.0001/SHEBNI.0001
1	120	N13-01301	NA	02-2012	Prepackaged chicken	2	NA	CP1	SHEXAI.0001/SHEBNI.0001
1	123	N13-01305	NA	02-2012	NDC	2	NA	CP1	SHEXAI.0001/SHEBNI.0001
1	130	N13-01315	NA	02-2012	Chicken sample	114	NA	CP1	SHEXAI.0114
1	131	N13-01316	NA	02-2012	Chicken sample	2	NA	CP1	SHEXAI.0001/SHEBNI.0001
1	132	N13-01317	NA	2012	Chicken sample	2	NA	CP1	SHEXAI.0001/SHEBNI.0001
1	144	N13-01338	NA	07-2012	Chicken sample	2	NA	CP1	SHEXAI.0001/SHEBNI.0001
1	148	N13-01349	NA	02-2012	Prepackaged chicken	2	NA	CP1	SHEXAI.0001/SHEBNI.0001
1	20	12-4179	Human	06-2012	NDC	2	29a	CP1	SHEXAI.0001/SHEBNI.0001
1	41	ID116157	Human	02-2012	NDC	2	19	CP1	SHEXAI.0001/SHEBNI.0001
2	85	ID119465	Human	08-2012	NDC	2	19	CP4	SHEXAI.0001/SHEBNI.0001
2	92	ID119981	Human	08-2012	NDC	2	19	CP4	SHEXAI.0001/SHEBNI.0001
2	95	ID120171	Human	09-2012	NDC	2	19	CP4	SHEXAI.0001/SHEBNI.0001
2	95	ID120227	Human	08-2012	NDC	2	19	CP4	SHEXAI.0001/SHEBNI.0001
3	23	12-5334	Human	07-2012	NDC	186	10	CP1	SHEXAI.0009/SHEBNI.0148
3	23	12-6342	Human	09-2012	NDC	52	19	CP1	SHEXAI.0009/SHEBNI.0025
3	23	12-7092	Human	09-2012	NDC	52	58	CP1	SHEXAI.0009/SHEBNI.0025
3	30	12-7327	Human	10-2012	NDC	52	19	CP1	SHEXAI.0009/SHEBNI.0025
3	141	N13-01332	NA	07-2012	Chicken sample	52	NA	CP1	SHEXAI.0009
3	143	N13-01337	NA	07-2012	Chicken sample	2	NA	CP1	SHEXAI.0001/SHEBNI.0001
4	67	ID118145	Human	05-2012	NDC	2	19	CP3	SHEXAI.0001/SHEBNI.0001
4	70	ID118312	Human	05-2012	NDC	2	19	CP3	SHEXAI.0001/SHEBNI.0001
4	70	ID119869	Human	08-2012	NDC	2	19	CP3	SHEXAI.0001/SHEBNI.0001
4	72	ID118450	Human	06-2012	NDC	2	19	CP3	SHEXAI.0001/SHEBNI.0001
4	72	ID121761	Human	11-2012	NDC	2	19	CP3	SHEXAI.0001/SHEBNI.0001
4	74	ID118629	Human	06-2012	NDC	2	NA	CP3	SHEXAI.0001/SHEBNI.0001
4	74	ID120975	Human	09-2012	NDC	2	19	CP3	SHEXAI.0001/SHEBNI.0001
4	74	N13-01323	NA	05-2012	Chicken cecal	2	NA	CP3	SHEXAI.0001/SHEBNI.0001
4	90	ID119898	Human	08-2012	NDC	2	19	CP3	SHEXAI.0001/SHEBNI.0001
4	135	N13-01324	NA	05-2012	Chicken cecal	2	NA	CP3	SHEXAI.0001/SHEBNI.0001
5	28	12-6507	Human	09-2012	NDC	52	29	CP2	SHEXAI.0009/SHEBNI.0025
5	49	ID116874	Human	03-2012	NDC	52	10	CP2	SHEXAI.0009/SHEBNI.0025
5	107	ID121807	Human	11-2012	NDC	2	19	CP2	SHEXAI.0001/SHEBNI.0001
5	151	N13-01353	NA	2012	Chicken sample	2	NA	CP2	SHEXAI.0001/SHEBNI.0001
6	2	12-0315	Human	01-2012	NDC	2	29	CP1	SHEXAI.0001/SHEBNI.0001
6	2	12-5335	Human	08-2012	NDC	2	29	CP1	SHEXAI.0001/SHEBNI.0001
6	2	N13-01295	NA	01-2012	Chicken sample	2	NA	CP1	SHEXAI.0001/SHEBNI.0001
6	2	ID119367	Human	07-2012	NDC	2	19	CP1	SHEXAI.0001/SHEBNI.0001
6	2	ID119818	Human	08-2012	NDC	2	19	CP1	SHEXAI.0001/SHEBNI.0001
6	75	ID118692	Human	06-2012	NDC	2	29	CP1	SHEXAI.0001/SHEBNI.0001
6	104	ID121444	Human	10-2012	NDC	2	19	CP1	SHEXAI.0001/SHEBNI.0001
7	3	12-0467	Human	01-2012	NDC	86	29	CP4	SHEXAI.0020/SHEBNI.0001
7	4	12-0469	Human	01-2012	NDC	86	29	CP4	SHEXAI.0020/SHEBNI.0001
7	26	12-5643	Human	08-2012	NDC	179	29	CP4	SHEXAI.0020
7	38	ID115709	Human	01-2012	NDC	86	29	CP4	SHEXAI.0020/SHEBNI.0001
7	46	ID116520	Human	02-2012	NDC	86	26a	CP4	SHEXAI.0020/SHEBNI.0001
7	52	ID117021	Human	04-2012	NDC	86	26	CP4	SHEXAI.0020/SHEBNI.0001
7	65	ID118102	Human	05-2012	NDC	165	41	CP4	SHEXAI.0020
7	101	ID120602	Human	09-2012	NDC	2	29	CP4	SHEXAI.0001/SHEBNI.0001
7	115	N13-01292	NA	2012	Chicken sample	86	NA	CP4	SHEXAI.0020
7	119	N13-01297	NA	2012	Chicken sample	86	NA	CP4	SHEXAI.0020
7	122	N13-01304	NA	02-2012	Prepackaged chicken	86	NA	CP4	SHEXAI.0020
7	134	N13-01321	NA	2012	Chicken sample	Unknown	NA	CP4	SHEXAI.0260
8	15	12-2695	Human	04-2012	04-2012-SH	2	19	CP1	SHEXAI.0001/SHEBNI.0001
8	15	12-3136	Human	04-2012	04-2012-SH	2	19	CP1	SHEXAI.0001/SHEBNI.0001
8	15	12-3327	Human	04-2012	04-2012-SH	2	NA	CP1	SHEXAI.0001/SHEBNI.0001
8	15	ID117237	Human	04-2012	04-2012-SH	2	19	CP1	SHEXAI.0001/SHEBNI.0001
8	15	ID117340	Human	04-2012	04-2012-SH	2	19	CP1	SHEXAI.0001/SHEBNI.0001
8	15	ID117349	Human	04-2012	04-2012-SH	2	19	CP1	SHEXAI.0001/SHEBNI.0001
8	15	ID119083	Human	07-2012	NDC	2	19	CP1	SHEXAI.0001/SHEBNI.0001
8	43	ID116364	Human	02-2012	NDC	2	19	CP1	SHEXAI.0001/SHEBNI.0001
8	48	ID116715	Human	03-2012	NDC	2	19	CP1	SHEXAI.0001/SHEBNI.0001
8	48	ID116933	Human	03-2012	NDC	2	19	CP1	SHEXAI.0001/SHEBNI.0001
8	48	ID117888	Human	05-2012	NDC	2	19	CP1	SHEXAI.0001/SHEBNI.0001
8	48	ID118035	Human	05-2012	NDC	2	19	CP1	SHEXAI.0001/SHEBNI.0001
8	48	ID118707	Human	06-2012	NDC	2	19	CP1	SHEXAI.0001/SHEBNI.0001
8	48	ID120599	Human	09-2012	NDC	2	19	CP1	SHEXAI.0001/SHEBNI.0001
8	53	ID117050	Human	04-2012	NDC	2	19	CP1	SHEXAI.0001/SHEBNI.0001
8	54	ID117315	Human	04-2012	NDC	4	24	CP1	SHEXAI.0011
8	56	ID117369	Human	04-2012	NDC	2	17	CP1	SHEXAI.0001/SHEBNI.0001
8	60	ID117841	Human	05-2012	NDC	4	41	CP1	SHEXAI.0011
8	60	ID117896	Human	05-2012	NDC	4	41	CP1	SHEXAI.0011
8	68	ID118190	Human	05-2012	NDC	2	19	CP1	SHEXAI.0001/SHEBNI.0001
8	88	ID119671	Human	08-2012	NDC	2	19	CP1	SHEXAI.0001/SHEBNI.0001
8	88	ID120945	Human	10-2012	NDC	2	19	CP1	SHEXAI.0001/SHEBNI.0001
8	96	ID120181	Human	09-2012	NDC	2	19	CP1	SHEXAI.0001/SHEBNI.0001
8	99	ID120509	Human	09-2012	NDC	2	29	CP1	SHEXAI.0001/SHEBNI.0001
8	126	N13-01311	NA	2012	Chicken cecal	2	NA	CP1	SHEXAI.0001/SHEBNI.0001
8	127	N13-01312	NA	2012	Chicken cecal	4	NA	CP1	SHEXAI.0011
9	8	12-1666	Human	02-2012	NDC	2	29	CP1	SHEXAI.0001/SHEBNI.0001
9	10	12-1847	Human	03-2012	NDC	52	29	CP1	SHEXAI.0009
9	10	N13-01318	NA	05-2012	Prepackaged chicken	52	NA	CP1	SHEXAI.0009
9	10	N13-01319Sa	NA	05-2012	Prepackaged chicken	52	NA	CP1	SHEXAI.0009
9	10	12-2554	Human	04-2012	NDC	2	18	CP1	SHEXAI.0001/SHEBNI.0001
9	51	ID116960	Human	03-2012	NDC	2	18	CP1	SHEXAI.0001/SHEBNI.0001
10	12	12-5634	Human	08-2012	NDC	2	19	CP1	SHEXAI.0001/SHEBNI.0001
10	12	12-2460	Human	03-2012	NDC	2	29	CP1	SHEXAI.0001/SHEBNI.0001
10	12	12-7145	Human	09-2012	NDC	2	19	CP1	SHEXAI.0001/SHEBNI.0001
10	12	ID115951	Human	01-2012	NDC	2	19	CP1	SHEXAI.0001/SHEBNI.0001
10	12	ID116766	Human	03-2012	NDC	2	29	CP1	SHEXAI.0001/SHEBNI.0001
10	12	ID118173	Human	05-2012	NDC	2	19	CP1	SHEXAI.0001/SHEBNI.0001
10	12	ID118688	Human	06-2012	NDC	2	19	CP1	SHEXAI.0001/SHEBNI.0001
10	12	ID115666	Human	01-2012	NDC	2	19	CP1	SHEXAI.0001/SHEBNI.0001
10	12	ID121948	Human	11-2012	NDC	2	19	CP1	SHEXAI.0001/SHEBNI.0001
10	29	12-6510	Human	09-2012	NDC	2	54	CP1	SHEXAI.0001/SHEBNI.0001
10	29	ID120288	Human	09-2012	NDC	2	54	CP1	SHEXAI.0001/SHEBNI.0001
10	47	ID116532	Human	02-2012	NDC	2	19	CP1	SHEXAI.0001/SHEBNI.0001
10	55	ID117342	Human	04-2012	NDC	2	19	CP1	SHEXAI.0001/SHEBNI.0001
10	57	ID117687	Human	05-2012	NDC	84	19	CP1	SHEXAI.0002
10	80	ID119047	Human	07-2012	NDC	2	29	CP1	SHEXAI.0001/SHEBNI.0001
10	84	ID121565	Human	11-2012	NDC	2	19	CP1	SHEXAI.0001/SHEBNI.0001
10	84	ID119198	Human	07-2012	NDC	2	19	CP1	SHEXAI.0001/SHEBNI.0001
10	102	ID120727	Human	09-2012	NDC	2	19	CP1	SHEXAI.0001/SHEBNI.0001
10	125	N13-01309	NA	05-2012	Prepackaged chicken	4	NA	CP1	SHEXAI.0011
10	137	N13-01326	NA	04-2012	Turkey sample	2	NA	CP1	SHEXAI.0001/SHEBNI.0001
10	142	N13-01336	NA	07-2012	Chicken sample	52	NA	CP1	SHEXAI.0009
11	113	N13-01290	NA	2012	Turkey sample	Unknown	NA	CP7	SHEXAI.0257
11	128	N13-01313	NA	2012	Turkey sample	Unknown	NA	CP7	SHEXAI.0257
12	140	N13-01331	NA	2012	Turkey sample	6	NA	CP1	SHEXAI.0111
12	154	N13-01366	NA	2012	Turkey sample	6	NA	CP1	SHEXAI.0111
13	27	12-6245	Human	08-2012	NDC	87	32	CP1	SHEXAI.0197
13	64	ID118044	Human	05-2012	NDC	87	32	CP1	SHEXAI.0197
13	133	N13-01320	NA	2012	Chicken sample	87	NA	CP1	SHEXAI.0197
14	106	ID121594	Human	11-2012	NDC	2	26	CP4a	SHEXAI.0001/SHEBNI.0001
14	106	ID121600	Human	11-2012	NDC	2	26	CP4a	SHEXAI.0001/SHEBNI.0001
14	106	ID121736	Human	11-2012	NDC	2	26	CP4a	SHEXAI.0001/SHEBNI.0001
15	5	12-1959	Human	02-2012	NDC	180	24	CP1	SHEXAI.0011
15	5	12-1016	Human	02-2012	NDC	180	41	CP1	SHEXAI.0011
16	32	12-7730	Human	10-2012	NDC	2	19	CP1	SHEXAI.0001/SHEBNI.0001
16	32	ID121207	Human	10-2012	NDC	2	19	CP1	SHEXAI.0001/SHEBNI.0001
NCC	19	12-3918	Human	06-2012	NDC	86	29	CP4	SHEXAI.0020
NCC	6	12-1063	Human	02-2012	NDC	86	29	CP4	SHEXAI.0020/SHEBNI.0001
NCC	6	12-3792	Human	05-2012	NDC	86	29	CP4	SHEXAI.0020/SHEBNI.0001
NCC	13	12-2552	Human	04-2012	NDC	2	29	CP6	SHEXAI.0001/SHEBNI.0001
NCC	24	ID121748	Human	11-2012	NDC	2	19	CP1	SHEXAI.0001/SHEBNI.0001
NCC	24	12-5542	Human	08-2012	NDC	2	17	CP1	SHEXAI.0001/SHEBNI.0001
NCC	24	ID119968	Human	08-2012	NDC	2	19	CP1	SHEXAI.0001/SHEBNI.0001
NCC	25	12-5632Sa	Human	08-2012	NDC	2	19	CP1	SHEXAI.0001/SHEBNI.0001
NCC	25	ID119990	Human	08-2012	NDC	2	19	CP1	SHEXAI.0001/SHEBNI.0001
NCC	31	12-7329	Human	10-2012	NDC	2	29	CP1	SHEXAI.0001/SHEBNI.0001
NCC	33	13-0067	Human	12-2012	NDC	52	29	CP1	SHEXAI.0009/SHEBNI.0025
NCC	37	ID115753	Human	01-2012	NDC	2	17	CP1	SHEXAI.0001/SHEBNI.0001
NCC	44	ID116979	Human	03-2012	NDC	2	19	CP1	SHEXAI.0001/SHEBNI.0001
NCC	50	ID116953	Human	03-2012	NDC	137	25	CP1	SHEXAI.0001
NCC	62	ID117887	Human	05-2012	NDC	181	4	CP4	SHEXAI.0141
NCC	66	ID118129	Human	05-2012	NDC	183	1	CP1	Not assigned
NCC	69	ID118298	Human	05-2012	NDC	52	Atypical	CP8	SHEXAI.0009/SHEBNI.0025
NCC	71	ID118349	Human	06-2012	NDC	2	19	CP1	SHEXAI.0001/SHEBNI.0001
NCC	73	ID118488	Human	05-2012	NDC	2	19	CP2	SHEXAI.0001/SHEBNI.0001
NCC	73	ID119541	Human	08-2012	NDC	2	19	CP2	SHEXAI.0001/SHEBNI.0001
NCC	77	ID118733	Human	06-2012	NDC	2	29	CP1	SHEXAI.0001/SHEBNI.0001
NCC	78	ID118983	Human	07-2012	NDC	2	19	CP1	SHEXAI.0001/SHEBNI.0001
NCC	79	ID119023	Human	07-2012	NDC	2	19	CP1	SHEXAI.0001/SHEBNI.0001
NCC	81	ID119099	Human	07-2012	NDC	2	19	CP9	SHEXAI.0001/SHEBNI.0001
NCC	89	ID119764	Human	08-2012	NDC	2	29	CP9	SHEXAI.0001/SHEBNI.0001
NCC	91	ID119947	Human	08-2012	NDC	2	Atypical	CP10	SHEXAI.0001/SHEBNI.0001
NCC	93	ID119993	Human	08-2012	NDC	2	19	CP1	SHEXAI.0001/SHEBNI.0001
NCC	94	ID120058	Human	08-2012	NDC	2	19	CP1	SHEXAI.0001/SHEBNI.0001
NCC	100	ID120587	Human	09-2012	NDC	2	19	CP1	SHEXAI.0001/SHEBNI.0001
NCC	105	ID121592	Human	11-2012	NDC	2	19	CP1	SHEXAI.0001/SHEBNI.0001
NCC	108	ID121903	Human	11-2012	NDC	2	19	CP1	SHEXAI.0001/SHEBNI.0001
NCC	109	ID121957	Human	11-2012	NDC	2	19	CP1	SHEXAI.0001/SHEBNI.0001
NCC	110	ID122078	Human	11-2012	NDC	2	19	CP1	SHEXAI.0001/SHEBNI.0001
NCC	111	ID122422	Human	12-2012	NDC	2	19	CP1	SHEXAI.0001/SHEBNI.0001
NCC	112	ID122529	Human	12-2012	NDC	2	19	CP1	SHEXAI.0001/SHEBNI.0001
NCC	117	N13-01294	NA	2012	Chicken sample	107	NA	CP2	SHEXAI.0201
NCC	121	N13-01303	NA	2012	Prepackaged chicken	2	NA	CP1	SHEXAI.0001/SHEBNI.0001
NCC	124	N13-01306	NA	2012	Prepackaged chicken	2	NA	CP1	SHEXAI.0001/SHEBNI.0001
NCC	129	N13-01314	NA	2012	Chicken sample	2	NA	CP1	SHEXAI.0001/SHEBNI.0001
NCC	136	N13-01325	NA	2012	Chicken cecal	2	NA	CP1	SHEXAI.0001/SHEBNI.0001
NCC	138	N13-01327	NA	2012	Turkey sample	Unknown	NA	CP1	SHEXAI.0116
NCC	139	N13-01330	NA	2012	Chicken cecal	52	NA	CP1	SHEXAI.0009
NCC	145	N13-01342	NA	2012	Chicken sample	52	NA	CP1	SHEXAI.0009
NCC	146	N13-01346	NA	2012	Chicken sample	4	NA	CP1	SHEXAI.0011
NCC	147	N13-01348	NA	2012	Chicken cecal	2	NA	CP1	SHEXAI.0001/SHEBNI.0001
NCC	149	N13-01351	NA	2012	Chicken sample	Unknown	NA	CP1	Not assigned
NCC	150	N13-01352	NA	2012	Chicken sample	4	NA	CP1	SHEXAI.0011
NCC	152	N13-01354	NA	2012	Turkey sample	86	NA	CP1	SHEXAI.0020
NCC	153	N13-01355	NA	2012	NDC	2	NA	CP5	SHEXAI.0001/SHEBNI.0001

*NCC, non-conclusive case; NDC, non-documented case; NA, non-available.*

### Whole Genome Sequencing

Isolates were cultured overnight at 37°C in brain heart infusion broth (BHIB). The genomic DNA was then extracted using the Metagenomic DNA isolation Kit for Water (Epicentre, Madison, WI, United States). Samples concentrations were measured with a Qubit fluorometer (Life Technologies, Carlsbad, CA, United States), standardized to 0.2 ng/μl and were stored at −20°C. Libraries were prepared using reagents provided in the Illumina Nextera XT DNA Library Preparation Kit (Illumina, Inc., San Diego, CA, United States) according to the manufacturer’s instructions. Paired-end sequencing was performed on the Illumina MiSeq system using 300 base read lengths. Whole-genome sequence contigs were *de novo* assembled using the SPAdes Genome Assembler integrated in IRIDA platform (Bankevich et al., 2012).

### Core Genome SNV (cgSNV) Analysis

cgSNV analysis was performed using the SNVPhyl pipeline v.1.0 (Petkau et al., 2017) which is integrated as an individual pipeline component within the NML galaxy system (Afgan et al., 2018). Briefly, SMALT v.0.7.5 (The Sanger Institute) was used to align paired-end sequence reads against *S*. Heidelberg SL476 reference genome (GenBank accession number NC_011083.1). MUMmer v.3.23 (Kurtz et al., 2004) and PHAST (Arndt et al., 2016) were used to identify repeat and prophage regions in the reference genomes, respectively, which were excluded from the analyses. FreeBayes v.0.9.20 (Garrison and Marth, 2012) and SAMtools (Li et al., 2009)/BCFtools (Minevich et al., 2012) calling algorithms were used to identify variants. The SNV alignment was run through PhyML to construct a maximum likelihood tree (Guindon et al., 2010) and FigTree v1.4 was used to generate dendrograms (The Institute of Evolutionary Biology, United Kingdom). PHYLOViZ v2.0 was used to construct the minimum spanning trees based on the geoBURST algorithm (Francisco et al., 2012).

### CRISPR Typing

A CRISPR type was defined by the unique spacer composition found in the two *Salmonella* CRISPR arrays, CRISPR1 and CRISPR2. The two *Salmonella* CRISPR loci, CRISPR1 and CRISPR2, were identified with the CRISPRFinder web service (Grissa et al., 2007). The direct repeat (29 nt) and spacer (32 nt) sequences were analyzed with Geneious and visualized with custom macros in Microsoft Excel. A CRISPR type of each isolate was defined as the CRISPR profile (CP) with a specific number reflecting its unique allelic type. The spacer sequence alignment was performed with Mega7 using Muscle. CRISPRTarget was used to identify protospacer matches. A match was defined as five or fewer SNPs between a spacer and a protospacer (Biswas et al., 2013; Shariat et al., 2015).

## Results

### Whole Genome Sequencing Results

We obtained an estimated average genome coverage of 99.4x (range, 30x–240.9x) for the set of 246 *S.* Heidelberg isolates. The number of SPAdes-assembled contigs (NrContigs) per isolate ranged from 17 to 256 but the majority (95.1%) of isolates assembled into fewer than 55 contigs (Supplementary Table S1).

### Cluster Detection Based on the cgSNV Analysis

**FIGURE 1:**
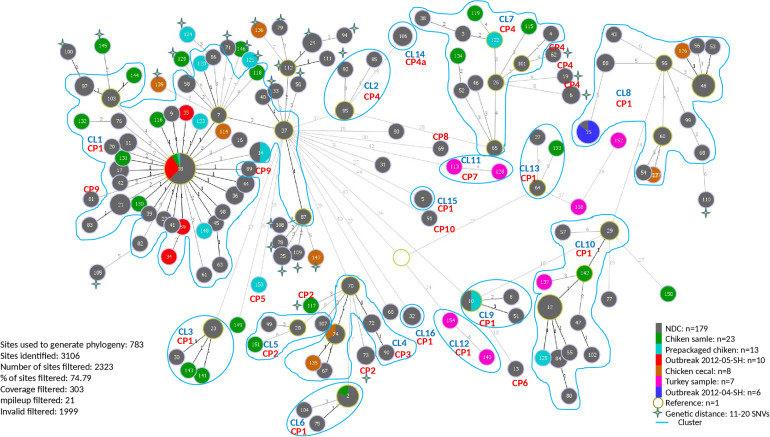
Minimum spanning phylogenetic tree based on the cgSNV analysis of 246 sequenced human and food *Salmonella enterica* serovar Heidelberg strains isolated in 2012 from the Province of Québec. The size of each node is proportional to the number of isolates and isolates in the same node have 0 SNV difference. The numbers in the circle represent the sequence types and the numbers on the branches connecting the circles represent the number of SNVs differences. CRISPR profiles (CP) are stated for each cluster (CL). The rest of the cgSNV sequence types displayed CP1. Non-documented cases (NDC) represent the isolates with incomplete epidemiological data.

A total of 154 sequence types (STs) were identified for the 246 *S*. Heidelberg isolates. The ST defines the set of isolates displaying a genetic distance of 0 SNV. The genetic distance interpretation was based on the Public Health Agency of Canada (PHAC)/PulseNet Canada guidelines used to interpret the relatedness of the outbreak isolates (0–10 SNVs). Based on the maximum likelihood (Supplementary Figure S1) tree and the minimum spanning tree analysis (Figure 1), 16 different clusters (CL), with at least two isolates in each cluster, were identified including clinical and/or food isolates. The genetic distances among each cluster was determined using similarity matrix (data not shown). The outbreak isolates were closely related to other isolates from the same outbreak based on the cgSNV analysis. The documented outbreaks belonged to two distinct clusters (CL1 and CL8) and the genetic distances observed within each outbreak was; 0 and 0–4 SNVs for the outbreak 2012-04-SH and the outbreak 2012-05-SH, respectively (Table 2).

**TABLE 2:** Genetic distances between isolates and potential sources of outbreaks and clusters using cgSNV/CRISPR analysis of the 246 *Salmonella enterica* serovar Heidleberg isolates (2012).

Outbreak/Cluster	Notes	Collection date (month)	No. of isolates	Number of SNV differences	CRISPR profile (CP)	Pulsotype (P) of isolates (human/food)	Potential source (s) /observation
05-2012-SH		05	10	0–4	CP1	P2 (*n* = 8/2)	
04-2012-SH		04	6	0	CP1	P2 (*n* = 6/0)	
CL1	- CL1 genetically related to outbreak 05-2012-SH - Outbreak 05-2012-SH is an UOB	01-10	89	0–10	CP1	P2 (*n* = 64/16), P4 (*n* = 3/0), P84 (*n* = 1/0), P114 (*n* = 0/1), NA = 4	- Chicken cecal (1 isolate), chicken sample (7 isolates) and prepackaged chicken (6 isolates). - 64 cases were probably part of the outbreak 05-2012-SH. - 2 isolates displaying CP9 were excluded from CL1
CL2	UOB (08-2012)	08-09	4	0–5	CP4	P2 (*n* = 4/0)	No source matches
CL3		07, 09,10	6	0–2	CP1	P2 (*n* = 0/1), P52 (*n* = 3/1), 186 (*n* = 1/0)	Chicken sample (2 isolates)
CL4	UOB (05-2012)	5,6,8,9,11	10	0–4	CP3	P2 (*n* = 8/2)	Chicken cecal (2 isolates)
CL5		03, 09, 11	4	0–9	CP2	P2 (*n* = 1/1), P52(*n* = 2/0)	Chicken sample (1 isolate)
CL6		01, 06–08, 10	7	0–4	CP1	P2 (*n* = 6/1)	Chicken sample (1 isolate)
CL7		01, 02, 04, 05, 08, 09	12	0–10	CP4	P2 (*n* = 1/0), P86 (*n* = 5/3), P165 (*n* = 1/0), 179 (*n* = 1/0), NA = 0/1	Chicken sample (3 isolates) and prepackaged chicken (1 isolate)
CL8	- CL8 genetically related to outbreak 04-2012-SH - Outbreak 04-2012-SH is an UOB - 2 UOB (05, 09-2012)	02–10	26	0–9	CP1	P2 (*n* = 21/1), P4 (*n* = 3/1)	- Chicken cecal (2 isolates) - 8 cases were probably part of the outbreak 04-2012-SH
CL9		02–04	6	0–3	CP1	P2 (*n* = 3/0), P52 (*n* = 1/2)	Prepackaged chicken (2 isolates)
CL10	3 UOB (05, 07, 09-2012)	01–09, 11	21	0–10	CP1	P2 (*n* = 17/1), P4 (*n* = 0/1), P52 (*n* = 0/1) P84 (*n* = 1/0)	Chicken sample (1 isolate), prepackaged chicken (1 isolate) and turkey sample (1 isolate)
CL11		NA	2	0–4	CP7	NA = 0/2	Cluster included turkey isolates only
CL12		NA	2	0–6	CP1	P6 (*n* = 0/2)	Cluster included turkey isolates only
CL13		05,08	3	0–10	CP1	P87 (*n* = 2/1)	Chicken sample (1 isolate)
CL14	UOB (11-2012)	11	3	0	CP4a	P2 (*n* = 3/0)	No source matches. Duplication of 5 bp on CRISPR1 (spacer 26)
CL15		02	2	0	CP1	P180 (*n* = 2/0)	No source matches
CL16		10	2	0	CP1	P2 (*n* = 2/0)	No source matches

*UOB, underestimated outbreak.*

In our study the PFGE patterns identified based on the guidelines described by Tenover et al. (1995) were confirmed to be genetically related by cgSNV as these isolates clustered together. Based on the epidemiological data, the categorization of isolates by cgSNV as outbreak-related was mostly concordant with the results obtained from the PFGE typing method. However, several isolates which were previously excluded from the outbreak investigation due to lack of epidemiological data were clustered with isolates of the two outbreaks as they differed by less than 10 SNVs, suggesting they may have been outbreak-related. Moreover, several human and food isolates displaying 11–20 SNVs were also probably related to the different clusters. Likewise, 10 putative distinct *S*. Heidelberg outbreaks, occurred in 2012, which were likely underestimated using PFGE analysis were categorized in separate clusters by our analysis. Additionally, cgSNV identified several clinical isolates (ST: 13, 77, 91, 31, 93, 69) as potential sporadic cases not related to the outbreaks and clusters (Figure 1). These isolates differed by >?20 SNVs from outbreak isolates and clusters which were not discriminated using PFGE method.

### CRISPR Profiles Distribution

**FIGURE 2:**
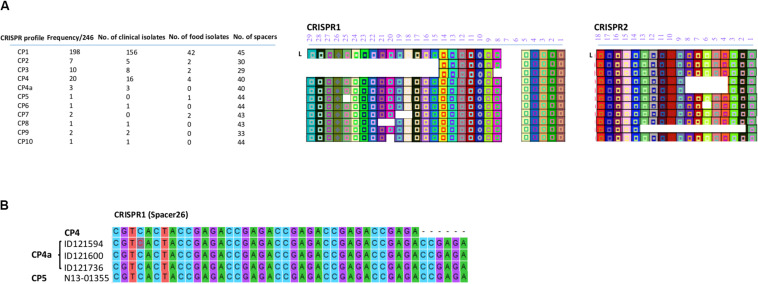
**(A)** CRISPR patterns and organization of spacer content of CRISPR alleles identified in the 246 human and food *S*. Heidelberg isolates. Each unique spacer is represented by a colored box and a symbol. The shape of the symbol indicates the length of the spacer. A change in the shape of symbol in CP4a within spacer 26 of CRISPR1 is due to repeated elements and signifies a change in the length of the spacer. The direct repeat sequences located between the spacers are not displayed. L is the position of the leader sequence. **(B)** Sequence alignment showing the repetition of 6 bp in spacer 26 located in the CRISPR1 array.

All the CRISPR1 and CRISPR2 arrays identified in this study are shown in Figure 2A. Based on the diversity of their spacer content, only 11 CRISPR profiles (CP) were identified. Putative last common ancestor (LCA), defined as an array containing a full complement of spacers (Shariat et al., 2015), harbored 29 unique spacers for CRISPR1 and 18 unique spacers for CRISPR2. None of the isolates displayed a LCA on the CRISPR1 as the number of spacers ranged from 11 to 27 and displayed eight different allelic types. On the other hand, the number of spacers in CRISPR2 ranged from 6 to 18 and exhibited four different allelic types. Duplication of spacers was not observed in any of the 246 *S*. Heidelberg isolates. This was concordant with the previous findings of Shariat et al. (2015) for the *S*. Heidelberg serovar.

Of note, two SNVs occurred in spacer 2 of CRISPR2 in the isolates classified in CP1, A-G and A-T of ID121948 and ID117342, respectively (Supplementary Table S2). All these isolates also belonged to CL10. However, these spacer SNVs were not taken into consideration to distinguish these isolates. Furthermore, we found a repetition of 6 bp (ccgaga) in spacer 26 located on CRISPR1 of the three isolates belonging to the CL14 (ID121594, ID121600, and ID121736) exhibiting CP4a and one food isolate (N13-01355) belonging to the CP5 (Figure 2B). This indicates that the spacer analysis may have some usability to help distinguish some clusters although this hypothesis requires a more-in depth investigation.

We also tried to determine whether any of the analyzed spacers matches phage or plasmid sequences (protospacers) using CRISPRTarget. Among the 396 arrays analyzed from the 246 isolates, only 6 spacers for which 14 putative protospacers were found; 5 spacers on CRISPR1 and 1 spacer on CRISPR2. Interestingly, only two protospacers were found in phage sequences (*Salmonella* phage SEN34 and Enterobacterial phage mEp39) while 12 protospacers were found in plasmid sequences (Supplementary Table S3).

Almost 80.5% (*n* = 198) of the isolates exhibited CRISPR profile 1 (CP1), containing 27 and 18 spacers in CRISPR1 and CRISPR2 arrays, respectively. Interestingly, this profile was the most observed in both clinical (156/193 isolates: 80.8%) and food isolates (42/53 isolates: 79.25%). CP1 was shared by both outbreaks (2012-05-SH, 2012-04-SH) and was also found in the majority of the CL1 and CL8 isolates, which are genetically related to the outbreaks. Our analysis revealed two NDCs [ID119099 (ST81) and ID119764 (ST89)], differentiated from CL1 based on CRISPR profile, exhibiting CP9 which lost 12 spacers in the CRISPR2 locus compared to CP1. The arrangement and microevolution of CRISPR spacers allows typing and subtyping. The high-resolution of CRISPR-based typing methods could constitute a practical means for rapid typing and source tracking. The CP4 was the second most frequent CRISPR profile at 8.3% (16/193) of clinical isolates and 7.6% (4/53) of food isolates. This profile lost 5 spacers (4–8) from the CRISPR2 locus and was found in two clusters (CL2 and CL7) and four other cases displaying ST62, 19 and 6 with 9–15 SNVs probably related to CL7. Cluster 4 and cluster 5, which were genetically close (2–11 SNVs differences), were also distinguished based on their CRISPR profiles. They displayed CP3 and CP2 for CL4 and CL5, respectively. In particular, CP5 and CP7 were only found in one prepackaged chicken (N13-01355) and in CL11 containing two retail turkey isolates (N13-01313 and N13-01290), respectively. Whereas, CP6, CP8, and CP10 were identified in sporadic clinical cases (12-2552, ID118298 and ID119947) genetically unrelated to any of the identified clusters.

### Source Tracking Using Combined cgSNV and CRISPR Analysis

Our analysis revealed that cgSNV typing linked outbreaks and different cluster isolates to their potential contaminating food source (s). The two isolates obtained during the food poisoning incidents which were obtained from food leftovers recovered from a marriage banquet were perfectly clustered with 2012-05-SH outbreak and several (64 isolates) other NDCs (0–10 SNVs differences). Interestingly, eight retail samples, six prepackaged and one abattoir chicken isolates were seen to have potential genetic linkages with 2012-05-SH outbreak and several cases which were part of the largest cgSNV cluster (CL1) in this study. Moreover, using WGS data, we also observed that the isolates genomically related belonged to the same CRISPR profile (CP1) as the outbreak isolates except for the two NDC displaying CP9 which were excluded from this cluster (Figure 1). Two chicken cecal isolates clustered with CL8, which is genetically related to 2012-04-SH outbreak displaying CP1. Two other chicken cecal isolates were also clustered with CL4 and displayed the same CRISPR profiles (CP3) as the clinical isolates. Among the clusters sharing CP4, only CL7 genetically linked to food isolates (one prepackaged chicken and three retail chicken sample isolates.) Retail chicken was the only possible source of CL3, CL5, and CL6. The prepackaged chicken was also the only potential source of the CL9. Interestingly, CL10 was associated to retail chicken and retail turkey food which indicates that the food contamination source could be due to multiple animal sources. Our analysis suggests poultry products and their environment as one potential source of *S.* Heidelberg infections.

## Discussion

The overarching goal of this research was to demonstrate how an integrative approach between stakeholders led to the identification of the potential source of the *S*. Heidelberg outbreaks that occurred in 2012 in Québec, Canada. Unlike PFGE method, cgSNV typing was highly discriminatory for *Salmonella* surveillance and outbreak support (Bekal et al., 2016; Vincent et al., 2018). Moreover, CRISPR typing could reveal genetic relatedness between strains or serovars and could be used for source tracking of *Salmonella* outbreaks (Deng et al., 2015; Xie et al., 2017). In this study, we analyzed clinical and food isolates, including chicken and turkey products, collected in Quebec in the same year as the outbreaks. The outbreaks and the majority of the human isolates analyzed exhibited pulsovar 2 based on PFGE analysis, while they were well separated into different unrelated clusters using cgSNV and CRISPR analysis. *S*. Heidelberg isolates within the outbreaks exhibited 0–4 SNVs differences between each other while the nearest NDCs to these outbreak groups differed by 0–10 SNVs. Until recently, it was difficult to link all the outbreaks cases using PFGE. Our findings suggest that the cgSNV analysis found NDCs that can probably be part of these outbreaks. Additionally, the analysis of different clusters showed that number of outbreaks were probably underestimated when using traditional typing methods because the epidemiological evidence to link these isolates was not available.

Investigation of spacer diversity revealed 11 different CRISPR profiles. The majority of the identified cgSNV clusters displayed CP1 and the remaining clusters exhibited different CRISPR profiles except for those sharing CP4. Our findings revealed that CP1 and CP4 might represent the predominant CRISPR type circulating among human and poultry *S*. Heidelberg isolates in 2012. It is tempting to suggest that these CRISPR types may serve as a guide for future prevention and surveillance programs. Furthermore, the identification of CP1, CP2, CP3, and CP4 in both poultry products and human isolates demonstrates the probable transmission of strains carrying these CRISPR types from poultry to human. No significant association between food type and CRISPR profile was observed, likely due to the fact that the analyzed isolates provided came from one source type (poultry). Previous study on *Salmonella enterica* serovar Enteritidis showed that different CRISPR profiles may circulate between food from different animal sources (duck and pig) (Li et al., 2018). Further studies including different animal sources of *Salmonella* serovars are needed to elucidate this aspect.

We previously demonstrated that CRISPR typing alone was less discriminatory compared to cgSNV (Vincent et al., 2018). The presence of identical CRISPR profiles among food, outbreak, non-outbreak isolates and unrelated clusters confirms the limitation of CRISPR subtyping in the investigation of outbreaks and food source tracking. However, some NDCs, food isolates and clusters genetically related by cgSNV could be separated based on the CRISPR typing. Therefore, CRISPR analysis can be used as a complementary approach for not only *Salmonella* foodborne outbreaks subtyping, but also for food source tracking.

Rapidly linking clinical isolates and possible food sources, during epidemiological investigation of outbreaks, is critical to eradicate the source(s) of the outbreaks and thereby limit its impact (Barco et al., 2013; Pires et al., 2014). In the current study, the food history of the patients was not available to suggest any food sources for sampling and testing. Nonetheless, we have been able to trace the potential source of a 2012 epidemiologically well-characterized foodborne outbreak and NDCs involving *S*. Heidelberg in Quebec. The combined cgSNV/CRISPR approaches were able to match and exclude food isolates from different clinical isolates clusters. The clustering of isolates from humans and food sources implicated poultry products as source for human infections. Our analysis is consistent with the results obtained by CIPARS where the chicken sources accounted for the majority (81%) of *S.* Heidelberg isolates, and of these, 76% were from retail chicken meat (Canadian Integrated Program for Antimicrobial Resistance Surveillance [CIPARS], 2015). However, more studies on the food survey data are needed to confirm our speculation.

The transmission of *S.* Heidelberg isolates from an environmental source to a food product vehicle and ultimately to humans is possible as several abattoir isolates were genetically related (0–10 SNVs) to food and human isolates based on both cgSNV and CRISPR typing. This confirms that the poor food-handling can be an important factor of transmission and cross contamination. Most often, public health stakeholders attribute foodborne outbreaks to one animal source during outbreaks investigations. Our analysis, however, showed that cross contamination due to multiple animal sources may also occur during food poisoning incidents, this is the case of the turkey and chicken isolates identified in the CL10. This finding may also indicate a potential risk of infection from inadequately handled poultry products (Jain et al., 2008; Griffith, 2013). Our analysis also revealed that the same strain from the same cluster could be found in different poultry product types in a given year and may be recovered at varying time intervals. These findings not only suggest a high environmental stability of some *S.* Heidelberg isolates but also that common contamination sources along the food production chain may favor the circulation of any given isolate for a long period.

## Conclusion

The cgSNV method is a highly discriminatory method for the resolution of clusters and outbreak events and the attribution of these events to their respective contaminating sources. The faster CRISPR typing can be useful for source tracking as well as serve as a complimentary tool to the cgSNV analysis during source attribution. This multi-disciplinary and multi-jurisdictional approach underscores the importance of using an integrated surveillance for outbreak investigations and source attribution. Although our findings are based on a limited number of food sources, our study, however, provides a potential tool to help identify sources of foodborne *S*. Heidelberg outbreaks especially if they are correlated with epidemiological data.

## Data Availability Statement

The datasets generated for this study can be found in the PRJNA541551.

## Ethics Statement

This study uses strains isolated from humans and obtained in the context of provincial surveillance program. Laboratoire de Santé Publique du Québec did not require the study to be reviewed or approved by an ethics committee because strains are obtained routinely for surveillance purpose and their secondary use do not require ethical study.

## Author Contributions

SB, KY, and VU conceptualized and designed the work. KY and VU wrote the manuscript, conducted the data analysis, interpretation and explored the core of the topic. MM, DT, and SM contributed to the methodology of the work by providing PFGE and CRISPR data, respectively. EF provided bioinformatics support. SB, CB, FD-B, LG, and CN supervised the project and provided the administrative resources. All authors critically revised and approved the final version of the manuscript.

## Conflict of Interest

The authors declare that the research was conducted in the absence of any commercial or financial relationships that could be construed as a potential conflict of interest.
