# Supplementary material for: Source Tracking Based on Core Genome SNV and CRISPR Typing of Salmonella enterica Serovar Heidelberg Isolates Involved in Foodborne Outbreaks in Québec, 2012
Source: Front Microbiol. 2020 Jun 17;11:1317. doi: 10.3389/fmicb.2020.01317 (PMC7311582; doi:10.3389/fmicb.2020.01317)
Supplement: Supplementary file 1 [file Data_Sheet_1.PDF]

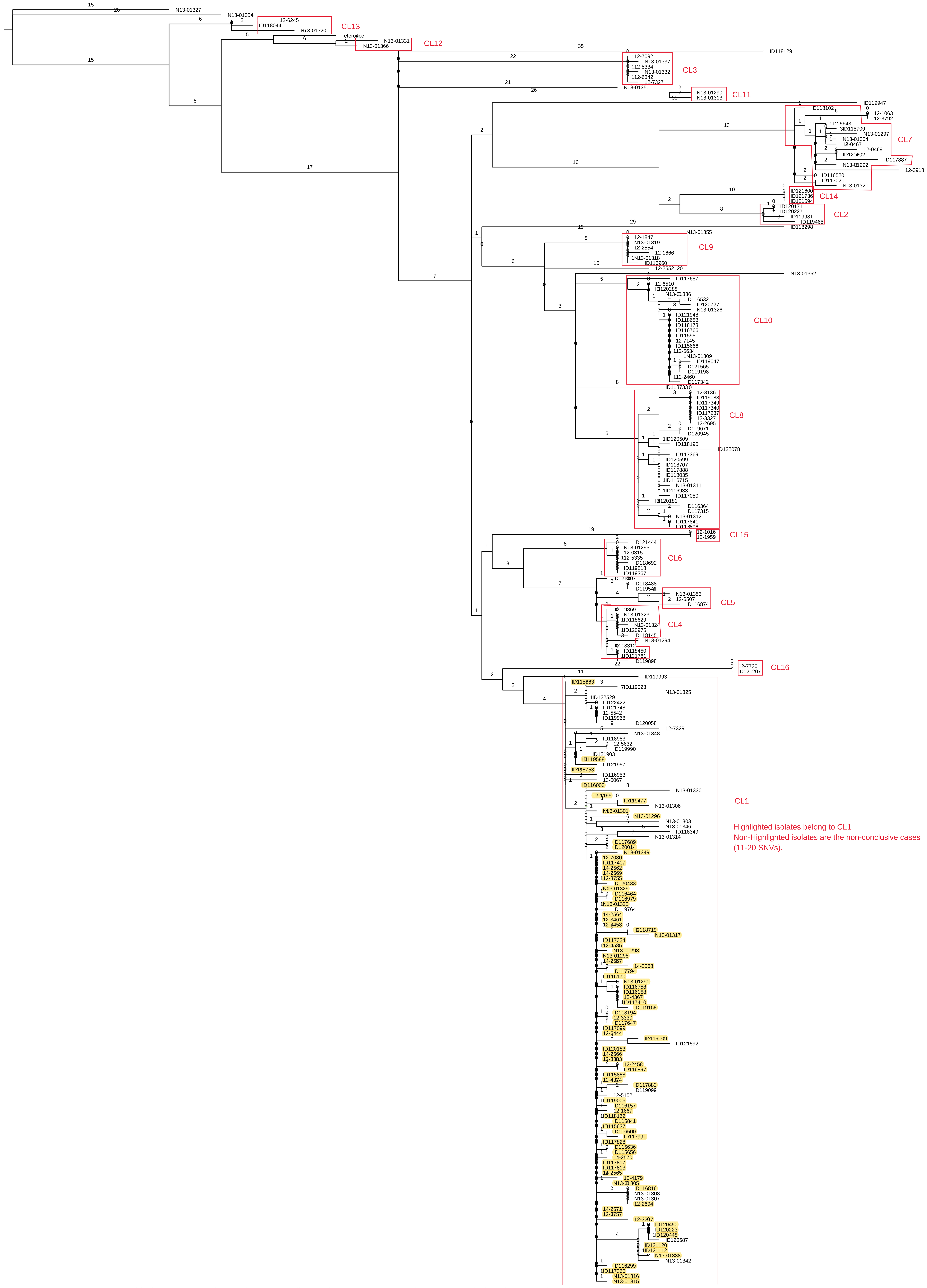

Figure S1: Maximum likelihood phylogenetic tree of 246 S. Heidelberg outbreak, non-outbreak and environmental isolates from QC collected in 2012.

9.0
