# Supplementary material for: Source Tracking Based on Core Genome SNV and CRISPR Typing of Salmonella enterica Serovar Heidelberg Isolates Involved in Foodborne Outbreaks in Québec, 2012
Source: Front Microbiol. 2020 Jun 17;11:1317. doi: 10.3389/fmicb.2020.01317 (PMC7311582; doi:10.3389/fmicb.2020.01317)
Supplement: Supplementary file 2 [file Table_1.DOCX]

**TABLE S1.** Coverage and assembly statistics for the 246 sequenced *S.* Heidelberg isolates used in this study.

| **Entry** | **N50** | **NrContigs** | **Length** | **Coverage** |
| --- | --- | --- | --- | --- |
| 12-0315 | 291737 | 32 | 4858414 | 86,8 |
| 12-0467 | 298793 | 47 | 4844405 | 94,5 |
| 12-0469 | 412159 | 37 | 4860588 | 63,8 |
| 12-1016 | 381668 | 28 | 4906069 | 61,4 |
| 12-1063 | 693678 | 27 | 4848337 | 62,6 |
| 12-1195 | 83829 | 123 | 4815505 | 92,7 |
| 12-1666 | 362963 | 27 | 4854634 | 109,1 |
| 12-1667 | 694160 | 24 | 4752083 | 57,8 |
| 12-1847 | 693405 | 24 | 4860970 | 153,3 |
| 12-1959 | 412162 | 27 | 4907333 | 84,2 |
| 12-2458 | 694160 | 26 | 4852901 | 91,3 |
| 12-2460 | 411864 | 27 | 4854668 | 54,4 |
| 12-2552 | 138863 | 86 | 4843179 | 119,4 |
| 12-2554 | 411931 | 28 | 4856586 | 109,1 |
| 12-2694 | 692615 | 29 | 4855644 | 119,9 |
| 12-2695 | 412096 | 25 | 4755028 | 132,9 |
| 12-3136 | 412030 | 26 | 4749935 | 71 |
| 12-3227 | 362964 | 27 | 4749786 | 81,7 |
| 12-3327 | 95135 | 81 | 4732904 | 77,5 |
| 12-3330 | 694160 | 23 | 4751400 | 106,4 |
| 12-3383 | 412096 | 24 | 4751682 | 109,3 |
| 12-3458 | 694160 | 23 | 4749247 | 97,0 |
| 12-3461 | 694160 | 23 | 4751788 | 151,7 |
| 12-3755 | 412096 | 28 | 4889780 | 84,5 |
| 12-3757 | 439725 | 24 | 4851532 | 155,2 |
| 12-3792 | 88673 | 104 | 4817695 | 112,5 |
| 12-3918 | 468053 | 23 | 4851275 | 136,5 |
| 12-4179 | 362964 | 27 | 4850691 | 162,1 |
| 12-4367 | 439725 | 23 | 4754055 | 78,2 |
| 12-4374 | 725308 | 23 | 4954378 | 130,6 |
| 12-4585 | 725312 | 24 | 4954815 | 111,3 |
| 12-5152 | 714539 | 26 | 4851993 | 134,8 |
| 12-5334 | 98952 | 87 | 4785361 | 86,8 |
| 12-5335 | 412162 | 26 | 4862945 | 156,3 |
| 12-5444 | 362964 | 28 | 4851166 | 106,6 |
| 12-5542 | 85009 | 105 | 4723953 | 92,9 |
| 12-5632 | 694941 | 23 | 4753263 | 99,9 |
| 12-5634 | 725307 | 21 | 4759968 | 128,0 |
| 12-5643 | 433235 | 26 | 4846839 | 90,1 |
| 12-6245 | 359001 | 30 | 4858709 | 186,2 |
| 12-6342 | 725305 | 23 | 4761514 | 157,0 |
| 12-6507 | 693240 | 26 | 4816772 | 126,2 |
| 12-6510 | 725307 | 22 | 4761290 | 171,7 |
| 12-7080 | 741300 | 19 | 4854846 | 190,0 |
| 12-7092 | 207535 | 48 | 4805011 | 95,9 |
| 12-7145 | 77572 | 123 | 4730368 | 75,5 |
| 12-7327 | 725301 | 20 | 4721380 | 151,0 |
| 12-7329 | 725308 | 23 | 4857970 | 119,5 |
| 12-7730 | 725306 | 21 | 4751436 | 166,0 |
| 13-0067 | 694154 | 25 | 4817184 | 158,0 |
| 14-2562 | 412162 | 24 | 4752626 | 124 |
| 14-2564 | 694160 | 24 | 4751706 | 82 |
| 14-2565 | 362809 | 24 | 4751129 | 178 |
| 14-2566 | 412030 | 25 | 4752289 | 101 |
| 14-2567 | 412096 | 23 | 4748953 | 59 |
| 14-2568 | 291966 | 30 | 4750290 | 92 |
| 14-2569 | 363274 | 25 | 4751226 | 82 |
| 14-2570 | 412030 | 25 | 4752108 | 73 |
| 14-2571 | 412162 | 24 | 4752907 | 85 |
| ID115636 | 412162 | 26 | 4751018 | 66 |
| ID115637 | 412030 | 27 | 4752422 | 94 |
| ID115656 | 248767 | 33 | 4844533 | 73 |
| ID115663 | 412162 | 29 | 4863702 | 84 |
| ID115666 | 412029 | 31 | 4755928 | 62 |
| ID115709 | 240064 | 33 | 4845712 | 96 |
| ID115753 | 412162 | 28 | 4863828 | 58 |
| ID115841 | 412162 | 25 | 4752277 | 120 |
| ID115858 | 363119 | 25 | 4751153 | 108 |
| ID115951 | 298840 | 31 | 4841210 | 131 |
| ID116003 | 412120 | 25 | 4751254 | 109 |
| ID116157 | 291966 | 30 | 4751033 | 116 |
| ID116158 | 411964 | 30 | 4751078 | 159 |
| ID116170 | 412162 | 25 | 4751026 | 144 |
| ID116299 | 693396 | 26 | 4752867 | 105 |
| ID116364 | 694175 | 27 | 4759200 | 101 |
| ID116464 | 411898 | 25 | 4752517 | 91 |
| ID116500 | 299025 | 28 | 4750462 | 137 |
| ID116520 | 298839 | 30 | 4744925 | 95 |
| ID116532 | 363118 | 27 | 4754544 | 105 |
| ID116715 | 251595 | 39 | 4749641 | 151 |
| ID116758 | 694941 | 23 | 4752548 | 122 |
| ID116766 | 291737 | 29 | 4854173 | 107 |
| ID116816 | 291734 | 32 | 4855481 | 75 |
| ID116874 | 276234 | 40 | 4716553 | 41 |
| ID116897 | 412162 | 25 | 4850812 | 84 |
| ID116933 | 291737 | 29 | 4749301 | 85 |
| ID116953 | 411964 | 26 | 4859618 | 66 |
| ID116960 | 291969 | 29 | 4750639 | 93 |
| ID116979 | 412030 | 29 | 4750859 | 76 |
| ID117021 | 412060 | 29 | 4742407 | 73 |
| ID117050 | 241153 | 36 | 4746022 | 68 |
| ID117099 | 276419 | 28 | 4854693 | 76 |
| ID117237 | 298621 | 30 | 4866751 | 78 |
| ID117315 | 412096 | 28 | 4889332 | 54 |
| ID117324 | 396675 | 30 | 4750390 | 81 |
| ID117340 | 363118 | 26 | 4748257 | 98 |
| ID117342 | 298840 | 27 | 4753689 | 49 |
| ID117349 | 412030 | 30 | 4748995 | 75 |
| ID117366 | 412105 | 30 | 4749537 | 91 |
| ID117369 | 276234 | 28 | 4748606 | 91 |
| ID117407 | 412030 | 26 | 4750479 | 79 |
| ID117410 | 291734 | 29 | 4748807 | 80 |
| ID117647 | 412162 | 28 | 4751000 | 86 |
| ID117687 | 298840 | 30 | 4844763 | 119 |
| ID117689 | 291734 | 30 | 4755183 | 133 |
| ID117794m | 362805 | 25 | 4750235 | 174 |
| ID117813 | 412162 | 25 | 4751364 | 119 |
| ID117817 | 412162 | 26 | 4751151 | 112 |
| ID117828 | 248739 | 31 | 4748853 | 62 |
| ID117841 | 362421 | 26 | 4886492 | 63 |
| ID117882 | 363119 | 29 | 4751482 | 51 |
| ID117887 | 298839 | 34 | 4817619 | 61 |
| ID117888 | 363118 | 29 | 4747178 | 67 |
| ID117896 | 291737 | 31 | 4886818 | 81 |
| ID117991 | 291966 | 29 | 4751200 | 83 |
| ID118035 | 412030 | 30 | 4749786 | 93 |
| ID118044 | 292130 | 38 | 4859767 | 41 |
| ID118102 | 270095 | 35 | 4882681 | 103 |
| ID118129 | 420846 | 30 | 4714658 | 51 |
| ID118145 | 412030 | 30 | 4762720 | 59 |
| ID118162 | 291966 | 29 | 4750817 | 90 |
| ID118173 | 39516 | 256 | 4762638 | 58 |
| ID118190 | 412162 | 25 | 4746295 | 51 |
| ID118194 | 247764 | 33 | 4752114 | 51 |
| ID118298 | 412163 | 30 | 4742854 | 117 |
| ID118312 | 412096 | 26 | 4761866 | 58 |
| ID118349 | 363119 | 25 | 4750592 | 199 |
| ID118450 | 381602 | 30 | 4760078 | 53 |
| ID118488 | 250601 | 31 | 4754600 | 60 |
| ID118629 | 288743 | 32 | 4760194 | 59 |
| ID118688 | 291443 | 42 | 4752882 | 50 |
| ID118692 | 694160 | 24 | 4851598 | 57 |
| ID118707 | 291969 | 27 | 4748736 | 52 |
| ID118719 | 411886 | 27 | 4750788 | 56 |
| ID118733 | 276235 | 31 | 4850214 | 50 |
| ID118983 | 363274 | 27 | 4751397 | 43 |
| ID119006 | 291934 | 34 | 4750896 | 65 |
| ID119023 | 412030 | 28 | 4752581 | 47 |
| ID119047 | 298840 | 31 | 4852801 | 62 |
| ID119083 | 276257 | 30 | 4748355 | 128 |
| ID119099 | 363274 | 25 | 4752242 | 103 |
| ID119109 | 276419 | 29 | 4751087 | 56 |
| ID119158 | 253443 | 31 | 4747119 | 41 |
| ID119198 | 298840 | 32 | 4849581 | 40 |
| ID119367 | 298839 | 30 | 4747984 | 30 |
| ID119465 | 247764 | 37 | 4747907 | 39 |
| ID119477 | 291966 | 28 | 4752142 | 76 |
| ID119541 | 362808 | 29 | 4750962 | 47 |
| ID119588 | 251602 | 31 | 4865358 | 75 |
| ID119671 | 381602 | 27 | 4752524 | 87 |
| ID119764 | 299025 | 29 | 4862968 | 71 |
| ID119818 | 298839 | 30 | 4749419 | 30 |
| ID119869 | 363118 | 29 | 4758475 | 85 |
| ID119898 | 276234 | 33 | 4761091 | 106 |
| ID119947 | 162220 | 56 | 4791757 | 76 |
| ID119968 | 276234 | 28 | 4751464 | 51 |
| ID119981 | 276233 | 28 | 4745403 | 54 |
| ID119990 | 275975 | 27 | 4749865 | 66 |
| ID119993 | 291961 | 31 | 4756203 | 77 |
| ID120014 | 276419 | 27 | 4751022 | 110 |
| ID120058 | 248767 | 46 | 4756496 | 51 |
| ID120171 | 362429 | 24 | 4746734 | 110 |
| ID120181 | 41005 | 245 | 4757966 | 59 |
| ID120183 | 276419 | 31 | 4752410 | 38 |
| ID120223 | 276268 | 26 | 4747374 | 65 |
| ID120227 | 298839 | 27 | 4749443 | 106 |
| ID120288 | 291737 | 30 | 4755631 | 123 |
| ID120433 | 298629 | 34 | 4846871 | 89 |
| ID120448 | 299025 | 29 | 4748874 | 91 |
| ID120450 | 291730 | 29 | 4748459 | 91 |
| ID120509 | 298840 | 29 | 4846641 | 91 |
| ID120587 | 252767 | 32 | 4748751 | 77 |
| ID120599 | 189385 | 46 | 4748641 | 42 |
| ID120602 | 276233 | 37 | 4841582 | 74 |
| ID120727 | 156693 | 55 | 4752389 | 45 |
| ID120945 | 412030 | 27 | 4783641 | 98 |
| ID120975 | 412162 | 27 | 4764236 | 89 |
| ID121112 | 291730 | 30 | 4747209 | 87 |
| ID121120 | 412162 | 23 | 4749636 | 99 |
| ID121207 | 298840 | 29 | 4748117 | 85 |
| ID121444 | 291737 | 31 | 4746791 | 59 |
| ID121565 | 412161 | 25 | 4752021 | 80 |
| ID121592 | 298599 | 35 | 4749240 | 86 |
| ID121594 | 276234 | 30 | 4746316 | 94 |
| ID121600 | 298840 | 28 | 4744840 | 78 |
| ID121736 | 412159 | 29 | 4749422 | 75 |
| ID121748 | 412035 | 29 | 4749527 | 98 |
| ID121761 | 363118 | 28 | 4758363 | 89 |
| ID121807 | 298840 | 35 | 4751588 | 41 |
| ID121903 | 298795 | 28 | 4748488 | 50 |
| ID121948 | 55272 | 214 | 4767589 | 58 |
| ID121957 | 291734 | 31 | 4750488 | 89 |
| ID122078 | 291737 | 41 | 4809205 | 53 |
| ID122422 | 363119 | 27 | 4746215 | 53 |
| ID122529 | 291966 | 29 | 4751722 | 70 |
| N13-01290 | 574055 | 32 | 5108977 | 144,3 |
| N13-01291 | 525573 | 25 | 4751319 | 85,9 |
| N13-01292 | 362963 | 27 | 4845267 | 135,8 |
| N13-01293 | 411964 | 26 | 4751469 | 164,0 |
| N13-01294 | 686046 | 24 | 4715865 | 104,1 |
| N13-01295 | 694160 | 25 | 4851420 | 94,4 |
| N13-01296 | 725307 | 20 | 4752909 | 134,1 |
| N13-01297 | 412159 | 31 | 4848161 | 81,5 |
| N13-01298 | 412096 | 25 | 4754731 | 55,1 |
| N13-01301 | 362964 | 25 | 4750608 | 121,1 |
| N13-01303 | 725308 | 20 | 4852157 | 240,9 |
| N13-01304 | 693855 | 33 | 4860111 | 101,1 |
| N13-01305 | 412162 | 24 | 4752182 | 113,7 |
| N13-01306 | 411964 | 24 | 4751662 | 107,0 |
| N13-01307 | 725312 | 25 | 4857558 | 137,4 |
| N13-01308 | 362964 | 30 | 4856340 | 145,1 |
| N13-01309 | 725307 | 22 | 4900123 | 117,2 |
| N13-01311 | 439723 | 20 | 4751094 | 169,7 |
| N13-01312 | 725299 | 23 | 4825664 | 185,5 |
| N13-01313 | 577253 | 43 | 5112371 | 102,1 |
| N13-01314 | 412030 | 26 | 4849146 | 150,9 |
| N13-01315 | 725308 | 22 | 4852847 | 169,1 |
| N13-01316 | 412096 | 30 | 4861676 | 116,8 |
| N13-01317 | 694047 | 24 | 4751951 | 132,9 |
| N13-01318 | 412030 | 28 | 4858926 | 107,8 |
| N13-01319 | 725307 | 20 | 4860565 | 133,5 |
| N13-01320 | 672257 | 30 | 4858405 | 163,2 |
| N13-01321 | 694629 | 24 | 4849239 | 80,9 |
| N13-01322 | 725307 | 23 | 4759533 | 105,7 |
| N13-01323 | 725307 | 22 | 4754460 | 167,9 |
| N13-01324 | 725230 | 22 | 4755506 | 135,5 |
| N13-01325 | 412096 | 26 | 4754545 | 157,8 |
| N13-01326 | 412095 | 25 | 4851407 | 100,4 |
| N13-01327 | 248767 | 36 | 4909811 | 148,7 |
| N13-01329 | 689216 | 22 | 4752999 | 134,1 |
| N13-01330 | 725308 | 21 | 4755769 | 156,6 |
| N13-01331 | 215270 | 39 | 4861968 | 135,7 |
| N13-01332 | 439725 | 18 | 4713512 | 145,4 |
| N13-01336 | 412162 | 25 | 4859000 | 134,1 |
| N13-01337 | 725305 | 21 | 4717525 | 111,2 |
| N13-01338 | 725312 | 20 | 4753161 | 179,6 |
| N13-01342 | 399644 | 26 | 4857300 | 222,5 |
| N13-01346 | 725308 | 19 | 4792412 | 124,6 |
| N13-01348 | 740519 | 17 | 4754283 | 148,5 |
| N13-01349 | 725312 | 25 | 4853088 | 198,4 |
| N13-01351 | 381485 | 24 | 4790459 | 170,5 |
| N13-01352 | 725306 | 22 | 4896302 | 121,2 |
| N13-01353 | 725307 | 22 | 4760241 | 147,3 |
| N13-01354 | 439486 | 26 | 4864438 | 93,1 |
| N13-01355 | 412162 | 25 | 4851400 | 170,3 |
| N13-01366 | 356053 | 33 | 4867947 | 209,8 |
