# Supplementary material for: Source Tracking Based on Core Genome SNV and CRISPR Typing of Salmonella enterica Serovar Heidelberg Isolates Involved in Foodborne Outbreaks in Québec, 2012
Source: Front Microbiol. 2020 Jun 17;11:1317. doi: 10.3389/fmicb.2020.01317 (PMC7311582; doi:10.3389/fmicb.2020.01317)
Supplement: Supplementary file 3 [file Table_2.DOCX]

| **Spacer** | **CRISPR 1 CRISPR 2** | |
| --- | --- | --- |
| 1 | aggggcgttccgcagtcgacaagggctgaaaa | cgttcatcggcagcgtcacgcaatatgaagat |
| 2 | ggttaaccaggggtttttccccactatttcgc | gacgagttctggaaatggttagctgataaaga  gacgagttctggaaatggtttgctgataaaga  gacgggttctggaaatggttagctgataaaga |
| 3 | tgacgaggtgcgagcgatggtatcaaggccta | caggttatgcgcaaaaattaattcatattata |
| 4 | gcaacccattaattaactaagcagtaataaac | ttgatcgagagtgcgaagaggcagaacgggca |
| 5 | tctggttataacatcgcagcaaaatcaaaaga | tgacgctggtctataccggcaacgaacgcgac |
| 6 | Not detected | aattatttctgtggctggggtttcgattcgat |
| 7 | Not detected | atgccggaacgctgatggcgtttgacatgagc |
| 8 | ttcttgaatatgattgcgggtatatgtggata | cggaggatggaatatttccgaggctggcgatt |
| 9 | agccgtttccgctaaatacccccgcagtgatt | cagatcctcaacggtcaggctgtttagttcct |
| 10 | gacgcgttccagcgcacgttactcgatc | aacaggaacaggaaaaaaaagatttgtccggt |
| 11 | aataaggcgcggtgccaccctcggctttaatt | cgtcagcgcggtattgaggccggggaccgccc |
| 12 | atattcgccgctttccatttaccgaacgtaac | aaaaaacagaagaacggcaagcggcacctcaa |
| 13 | gtcgttcatcaggcactaccggcactttctgg | ccggcatcagcgccgatccgttcatagtgccc |
| 14 | tggattatctgtattttacggaagtgggcgcg | gcgaggtcaataaaaaatggtgtggctttacc |
| 15 | gtttgccgtatcttcgatcataccggaacggt | ttttgatacgtagtattcattacgcctcctag |
| 16 | acgccccgaatgtgtttgcctcgcccgctgcc | ctccagcgctcgaatttatttgaggccaccac |
| 17 | gaggcgtacaggctgttagatgagaaattacc | gcagcggttgagtaactcctcgtccacgtcga |
| 18 | ttacgtgtttattcatctgttgcattagattc | ttccagaaccgtttgacttactgtggccatta |
| 19 | cgtcgcggaaaatttcgcattgacgataaaga |  |
| 20 | ttgcagggcgatattgttgttggtgaatggga |  |
| 21 | ccgctgacgcactggatcaacctgacgcaacg |  |
| 22 | gcgcgccaataattttattgacgatttcatca |  |
| 23 | gcggctctgtgttgggcgatggctccggtggt |  |
| 24 | ttaaatccatatacgggccttgcgggtttgcc |  |
| 25 | cctttaatcgcctcttatcgcctggattggtt |  |
| 26 | cgtcactaccgagaccgagaccgagaccgagaccgaga  cgtcactaccgagaccgagaccgagaccgagaccgagaccgaga |  |
| 27 | cagtgagatgccgccaatttgtcaaataaaat |  |
| 28 | gctgggtagtggagtaatcattatgtgtggtg |  |
| 29 | gggaaaaatcaataaaatcaatgataagcagt |  |

**Table S2**. Spacer sequences found in CRISPR 1 and CRISPR 2 loci of the 246 *Salmonella enterica* serovar Heidelberg isolates. Mutations in the spacers highlighted in green.
